# Effect of Clinician Posture on Patient Perceptions of Communication in the Inpatient Setting: A Systematic Review

**DOI:** 10.1007/s11606-024-08906-4

**Published:** 2024-07-17

**Authors:** Nathan Houchens, Jason M. Engle, Rita Palanjian, Sanjay Saint, Whitney A. Townsend, Mariam Nasrallah, Ashwin Gupta

**Affiliations:** 1 Medicine Service, Veterans Affairs Ann Arbor Healthcare System, Ann Arbor, MI USA; 2 Department of Internal Medicine, University of Michigan Medical School, Ann Arbor, MI USA; 3 Center for Clinical Management Research, Veterans Affairs Ann Arbor Healthcare System, Ann Arbor, MI USA; 4 University of Michigan Medical School, Ann Arbor, MI USA; 5 Department of Urology, University of Arizona, Tucson, AZ USA; 6 University of Michigan Taubman Health Sciences Library, Ann Arbor, MI USA; 7 Beaumont Health Family Medicine, Wayne, MI USA

**Keywords:** provider posture, physician posture, sitting vs standing, patient experience, hospital medicine, quality of care

## Abstract

**Background:**

Nonverbal communication plays a pivotal role in the provision of effective patient care and has been associated with important patient health outcomes. Clinician posture, a nonverbal form of communication, may influence the patient experience and satisfaction. The relationship between clinician posture (i.e., standing or at the patient’s eye level) and patient perceptions of clinician communication in the hospital—a setting with heightened power dynamics between patient and clinician—is currently unknown.

**Methods:**

We conducted searches of Ovid MEDLINE, EBSCO CINAHL Complete, EBSCO PsycInfo, Elsevier Embase/Embase Classic, Elsevier Scopus, and Web of Science Core Collection up to May 2023. English language studies were included if they compared clinician posture (eye-level or standing) during adult inpatient (including emergency department) interactions. Two authors independently abstracted data from included studies and assessed risk of bias or quality of evidence. A third author arbitrated any disagreements. Studies reported adherence to the posture intervention and/or patient perception outcomes. The latter included encounter duration, preferences for posture type, perceptions of interaction quality and clinician communication and compassion, and standardized assessments of patient satisfaction.

**Results:**

Fourteen studies (six randomized controlled trials, four quasi-experimental studies, four observational studies) assessed clinician posture at the bedside. Ten noted at least one favorable outcome for clinicians who communicated at the patient’s eye level, three revealed no differences in patient perceptions between standing and sitting, and one noted higher patient ratings for standing clinicians. Findings were limited by variation in interventions and outcomes, generally high risk of bias, and relatively low adherence to assigned posture groups.

**Discussion:**

Compared to standing, eye-level communication by clinicians appears beneficial. The magnitude and types of benefits clinicians and patients may gain from this behavior remain unclear given heterogeneity and generally high risk of bias in available studies. With its relatively easy implementation and potential for benefit, clinicians should consider communicating with their hospitalized patients at eye level.

**Registration:**

PROSPERO, CRD42020199817.

**Supplementary Information:**

The online version contains supplementary material available at 10.1007/s11606-024-08906-4.

## INTRODUCTION

Communication in healthcare environments is complex, multi-dimensional, and dynamic.^1^ The style and quality of clinician communication may influence patient satisfaction,^2,3^ adherence to treatment plans,^4–7^ 30-day hospital readmission,^8^ information recall,^2^ emotional health,^9^ and health outcomes.^10,11^ While verbal communication strategies have historically garnered the most attention, nonverbal communication is increasingly recognized as an essential component in the provision of high-quality patient care and crucial to the patient-clinician relationship.^12–14^ Effective nonverbal interactions are associated with enhanced patient satisfaction, trust, confidence, rates of malpractice litigation, adherence, symptom resolution, and improvements in health outcomes.^2,3,13,15–23^

From choice of attire to facial expression to gestures, nonverbal communication is defined as behaviors that carry no linguistic content.^24^ Nonverbal communication can be an important vehicle for demonstrating compassion^19^ and has been described as “the channel most responsible for communicating attitudes, emotions, and affect.”^25^ It also conveys much about interpersonal interactions and status between two individuals. It can clarify verbal messages, modulate interactions, and relay attitudes such as support and interest in the other.^12,26,27^ Prior literature has shown that one can rapidly determine personality traits like dominance,^28^ social relationship type,^29^ and intentionality and motives^30^ using nonverbal communication cues alone. Hospitalization is a vulnerable period, because patients are often more acutely ill than those who are receiving outpatient care. As a result of their conditions, hospitalized patients often spend more time in bed, have less of a sense of personal agency and physical ability, and are more dependent on the health system. Thus, the hospital can be a setting in which the effects of power dynamics between patient and clinician, both overt and covert, are heightened beyond those experienced in other healthcare settings. Through their communication behaviors, clinicians may affect the sharing of power with patients.^31^

The impacts of clinician posture—i.e., standing at the bedside or seated at the patient’s eye level (hereafter referred to as “sitting”)—on outcomes like the patient experience and satisfaction with communication have shown conflicting results in the literature. This systematic review, therefore, aims to examine the relationship between clinician posture and patient perceptions of communication in the inpatient hospital setting.

## METHODS

### Protocol and Registration

We registered the protocol for this review with PROSPERO, the international prospective register of systematic reviews, on 21 August 2020 (CRD42020199817). We deviated slightly from our protocol, because assessed outcomes and study types differed from our original expectations.

### Data Sources and Searches

The following databases were searched from inception up to 5 May 2023 in order to identify relevant articles, trials, or meeting abstracts describing clinician posture in the inpatient setting: Ovid MEDLINE(R) and Epub Ahead of Print, In-Process, In-Data-Review & Other Non-Indexed Citations, Daily and Versions(R), Elsevier Embase (including Embase Classic), Elsevier Scopus, Web of Science Core Collection (SCI-EXPANDED; SSCI; A & HCI; CPCI-S; CPCI-SSH; BKCI-S; BKCI-SSH; ESCI; CCR-EXPANDED), EBSCO CINAHL Complete, and EBSCO PsycInfo. Each search utilized controlled vocabulary whenever possible in combination with relevant keywords. No limits were applied to the search. A set of seminal articles were identified before the search process and were used to generate search terms and test the effectiveness of the strategies in each database. Reference tracking was performed on highly relevant articles/included papers. Original search strategies were developed in Ovid MEDLINE and translated as appropriate to the other databases using the Systematic Review Accelerator Polyglot tool.^32^ Citations were deduplicated using a modified version of the Bramer Endnote Deduplication Technique.^33^ The first search was conducted on 1 October 2020. Repeat searches were conducted on 14 February 2022 and 5 May 2023 to identify recently published results, and we included those, as appropriate, in our final set of studies. The complete search strategies are available in Appendix 1.

### Study Selection

We utilized Covidence software to perform screening of studies returned by our systematic searches.^34^ After each search or search update, two authors (2020: MN, RP; 2022: RP, JME; 2023: JME, AG) used Covidence to include or exclude studies, and a third author (NH) resolved any conflicting selections. Studies were eligible for inclusion if they involved clinicians (any member of the healthcare team) and adult patients in the inpatient setting. For the purposes of this search, we considered studies conducted in the emergency department (ED) as “inpatient.” Studies of any design were included if they compared sitting clinicians to those standing during patient interactions, and if they were published in English. Study interventions may have included posture in isolation or as part of a broader intervention bundle. We included studies measuring adherence to the intervention, patient perspectives of communication (e.g., perceived clinician time spent with patient, posture preferences, clinician characteristics like compassion), or the results of standardized assessments of patient satisfaction (e.g., Hospital Consumer Assessment of Healthcare Providers and Systems [HCAHPS] and Press Ganey satisfaction surveys).

**Figure 1:**
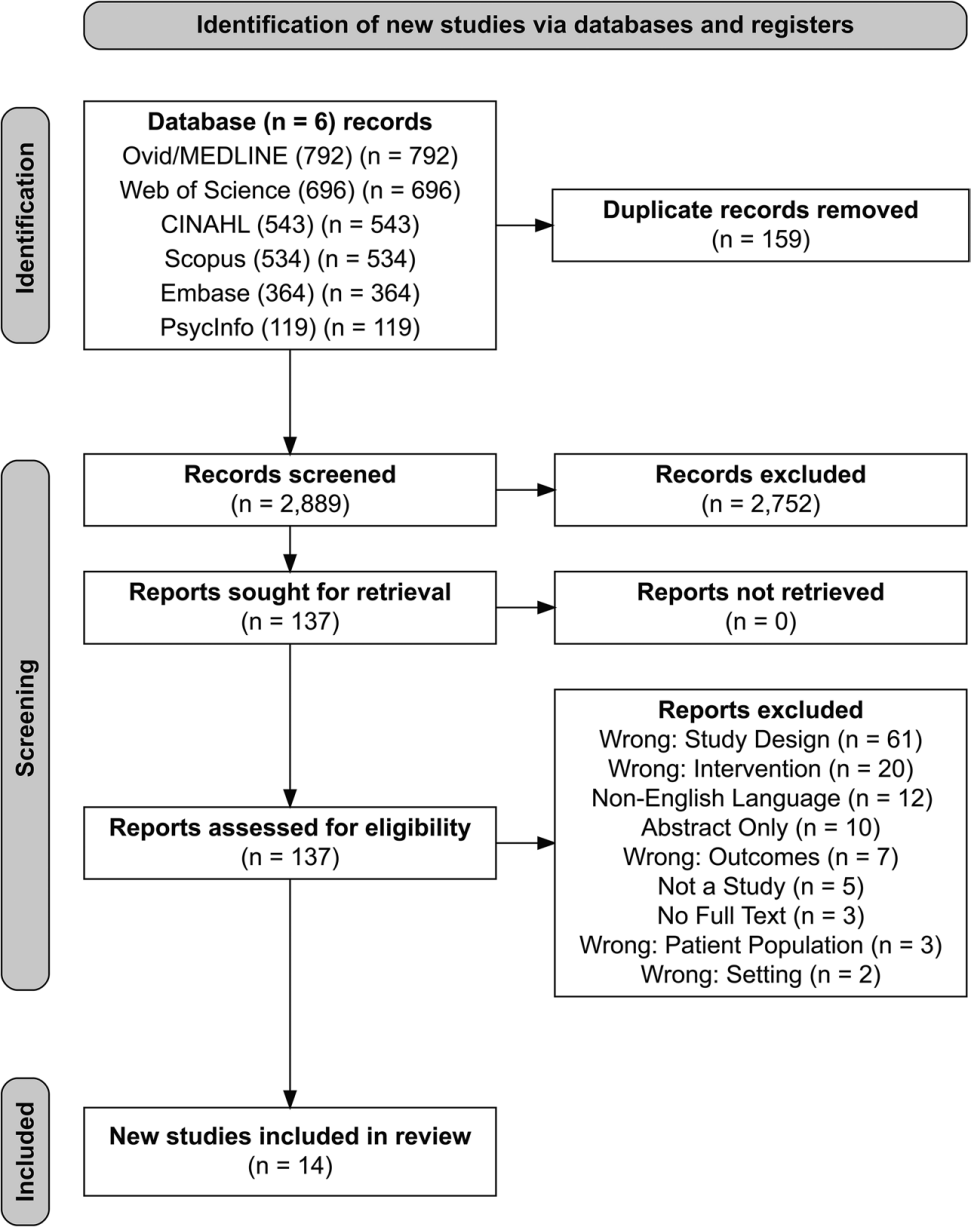
PRISMA diagram.

We excluded studies that were conducted in outpatient settings (i.e., ambulatory clinic visits) unless inpatients were explicitly recruited. Our searches included publications that were editorial or perspective in nature, but these were ultimately excluded due to insufficient study information. Finally, we excluded studies that were poster-only presentations and those that contained obvious exclusion criteria in the title. See Fig. 1 for the PRISMA diagram showing the selection process in full.^35^

### Data Extraction and Quality Assessment

We completed data extraction without significant deviations from the pre-defined protocol. Although we had proposed extracting recruitment and study completion rates, suggested mechanisms of intervention action, and times of measurement, these data were not available in the reviewed studies. We extracted the remaining data elements specified in the protocol using Covidence software; see Appendix 2 for the data extraction template we used. Two authors (RP, JME) performed independent data extractions, and one (NH) adjudicated disagreements. We contacted study corresponding authors for missing data, and while responsive, they were unable to provide the requested data.

We assessed risk of bias in randomized studies using version 2 of the Cochrane risk-of-bias tool for randomized studies (RoB 2).^36^ For quasi-experimental studies, we assessed using the Risk Of Bias In Non-randomized Studies–of Interventions (ROBINS-I) tool.^37^ To assess quality of evidence in observational studies, we used the Downs and Black checklist.^38^ Because these scales are not comparable to one another, we cannot draw definitive conclusions across study types; however, comparisons within study types are valid.

### Data Synthesis and Analysis

We analyzed studies based on a variety of assessed outcomes including adherence to assigned posture group, patient perceptions, and patient satisfaction survey results. Because we often could not compare quantitative outcomes for studies due to differences in study type or outcomes assessed, we present our analysis as a systematic-narrative hybrid review.^39^ This approach allows for presentation of the interventions and outcomes within each study’s unique context. Study heterogeneity also precluded the ability to conduct a meta-analysis.

### Role of the Funding Source

The authors did not receive any external funding for the submitted work.

## RESULTS

### Study Selection

We identified a total of 3048 citations in three searches conducted on 1 October 2020, 14 February 2022, and 5 May 2023. Of these, 2889 (94.8%) underwent title and abstract review, 137 (4.5%) underwent full text screening, and 14 (0.5%) met study inclusion criteria (Table 1). We also identified ten conference abstracts (see Appendix Table 2) in the final cohort; however, given their redundancy with–and paucity of information compared to–other included articles, we did not include these in the final analysis.

**Table 1:** Studies Included in this Systematic Review

Author, Year	RoB/QoE	Journal	Population	Intervention(s)	Outcome(s)	N*	Key Finding(s)	p*
Randomized controlled trials (risk of bias assessed via RoB 2^36^)								
Bruera, 2007^40^	Low	*Palliat Med*	Inpatient and outpatient	Randomized assignment of video (sit then stand or stand then sit)	Patient preference for sitting or standing clinician	168	Sitting preferred to and seen as more compassionate than standing	< 0.001
Donovan, 2020^41^	High	*Patient Educ Couns*	Inpatient	Randomized to sit or stand	Patient satisfaction (bespoke survey)	347	Standing clinicians rated higher in 2 of 5 communication domains; no differences between groups for the remaining 3 domains	0.037/0.021^**†**^ 0.032/0.004^‡^
Johnson, 2008^42^	High	*Ann Emerg Med*	Emergency Department	Randomized to sit or stand	Patient-reported time spent with clinician and quality of interaction	224	Patients with sitting clinicians overestimated time together, patients with standing clinicians underestimated time together	0.001
Merel, 2016^43^	High	*J Hosp Med*	Inpatient	Randomized to sit or stand	Patient perception of clinician communication skill	159	Patients perceived seated clinicians explained things better and listened more carefully	0.01
Strasser, 2005^44^	Low	*J Pain Symptom Manage*	Inpatient and outpatient	Randomized assignment of video (sit then stand or stand then sit)	Patient preference for sitting or standing clinician	69	Patient preferred posture/compassion rating favored second position observed	0.003/0.002
Swayden, 2012^45^	High	*Patient Educ Couns*	Inpatient	Randomized to sit or stand	Actual and patient-perceived time spent with clinician	120	Patients perceived clinicians spent more time with them when seated at their bedside	0.01
Quasi-experimental studies (risk of bias assessed via ROBINS-I^37^)								
George, 2018^46^	High	*Crit Care Nurse*	Inpatient	Sitting with patients 3–5 min to review plan of care for the day	Patient satisfaction (HCAHPS) including nurse communication	—	Sitting with patients improved overall HCAHPS scores	—
Horton, 2017^47^	Very high	*Am J Med Qual*	Inpatient	Standardized multi-component communication intervention	Patient satisfaction (HCAHPS) with clinician communication	3837	Clinician communication scores increased for intervention	< 0.001
Orloski, 2019^48^	Very high	*J Patient Exp*	Emergency Department	Educational campaign on good clinician communication	Patient satisfaction (bespoke survey)	2827	Patient satisfaction increased	< 0.001
				Placed chairs in ED bays	Frequency of clinician sitting		Odds of sitting increased 30% (OR 1.3 [CI 1.1–1.5])	—
Pattison, 2017^49^	Very high	*J Nurs Care Qual*	Inpatient	Randomized to sit or stand	Patient satisfaction (bespoke survey)	76	No differences between seated and standing groups	—
					Patient-perceived time with clinician		No differences between seated and standing groups	0.54
Observational (cross-sectional) studies (quality of evidence assessed via Downs and Black^38^)								
Golden, 2022^50^	Poor	*J Gen Intern Med*	Inpatient	N/A	Patient-reported frequency of clinician sitting	256	Frequent sitting correlated with positive behaviors (spending enough time at the bedside, checking for understanding, and not seeming to be in a rush)	< 0.001 (for all)
Gupta, 2015A^52^	Poor	*J Med Pract Manag*	Inpatient	N/A	Patient preference for sitting or standing clinician	127	Most patients preferred seated vs. standing clinicians	—
Gupta, 2015B^53^	Poor	*J Med Pract Manag*	Inpatient	N/A	Patient preference for sitting or standing clinician	85	Most patients preferred seated vs. standing clinicians	—
Tackett, 2013^51^	Poor	*J Gen Intern Med*	Inpatient	N/A	Observer-reported frequency of EtBM (including sitting)	226	Clinicians spending more time with patients more likely to perform EtBM	< 0.05
					Press-Ganey scores		Sitting/performing EtBM associated with higher scores	0.026/0.019

*EtBM* etiquette-based medicine behaviors, *HCAHPS* hospital consumer assessment of healthcare providers and systems, *QoE* quality of evidence, *RoB* risk of bias

^*^Dash in column indicates data are not available

^†^*p*-values represent intention-to-treat analysis/per-protocol analysis (respectively) comparing standing to sitting for the communication domain “Physician told me everything.”

^‡^*p*-values represent intention-to-treat analysis/per-protocol analysis (respectively) comparing standing to sitting for the communication domain “Physician gave me time to ask questions.”

### Study Characteristics

A total of six included studies were randomized controlled trials,^40–45^ four were quasi-experimental,^46–49^ and four were cross-sectional, observational studies.^50–53^ Per protocol, all studies either took place in or recruited patients from the inpatient or ED settings. Twelve studies included physicians.^40–45,47,48,50–53^ Of these, several examined more than one level of physician, with four including resident physicians,^41,42,48,50^ six including attending physicians,^42,43,45,47,48,51^ and four including physicians without level specification.^40,44,52,53^ Three studies included nurses,^46,48,49^ two included Advanced Practice Providers,^42,48^ and one included medical students.^42^ Studies were performed on medical/medical-surgical wards in ten instances,^41,43,45–47,49–53^ with two performed in the ED.^42,48^ The final two studies were performed in the outpatient setting^40,44^ and were included because they recruited hospitalized patients.

Ten studies measured the impact of posture alone,^40–45,48,50,52,53^ whereas four included clinician posture as part of an intervention bundle.^46–48,51^ Assessed outcomes varied across studies. Four studies directly observed clinician posture or actual time spent with patients,^42,43,45,51^ 13 reported patient preferences (e.g., overall satisfaction, perceived clinician posture, perceived clinician time spent with patient, posture preferences, or clinician characteristics like compassion),^40–50,52,53^ and four reported results of standardized assessments of patient perceptions including the Hospital Consumer Assessment of Healthcare Providers and Systems [HCAHPS] survey^41,46,47^ and Press Ganey satisfaction survey.^51^

### Risk of Bias Summary

Among randomized studies (*n* = 6), two had low risk of bias, while the remaining four had high risk of bias (Table 1). All quasi-experimental studies (*n* = 4) had a high or very high risk of bias. All observational studies (*n* = 4) had “poor” quality of evidence (conceptually similar to high risk of bias, but for the Downs and Black checklist). High risk of bias among randomized studies was most often due to non-adherence with the intervention (e.g., standing when assigned to sitting), whereas high risk of bias among quasi-experimental studies was most often due to lack of control for confounding variables.

## Results of Individual Studies

### Adherence to the Intervention

Four studies assessed observed or perceived adherence with individual postures. In the evaluation of ED clinicians assigned to stand or sit, Johnson found that adherence with the assigned standing and sitting groups was 100% and 67.4%, respectively.^42^ Similarly, in a study of senior resident clinicians, Donovan found adherence rates were 71.9% and 49.4% for residents randomized to standing and sitting, respectively.^41^ Merel examined hospitalists and reported rates of intervention adherence of 94% and 83% for standing and sitting, respectively; however, these rates were based on clinician self-report rather than direct observation.^43^ Tackett found that 0 of 6 etiquette-based medicine behaviors, which included sitting at the bedside, were performed by hospitalists on a majority of patients.^51^ Among the four studies that reported observed patient encounters, there were no differences in actual encounter times based on clinician posture.^42,43,45,51^

Two studies assessed patient perceptions of the frequency of clinicians’ chosen posture. In Orloski’s evaluation of ED patients, approximately 40% of patients reported that at least one clinician sat during the encounter.^48^ Similarly, in Golden’s survey study of hospitalized medical patients, 55.8% of patients reported that internal medicine resident clinicians “never” sat.^50^

### Patient Perception Outcomes

#### Encounter Duration

 In the evaluation of patient perceptions of encounter duration based on clinician posture, Johnson found that patients involved in sit-randomized interactions overestimated time clinicians spent with them by an average of 1.3 min (SD 4.3 min), whereas those involved in stand-randomized interactions underestimated time by 0.6 min (SD 4.3 min), *p* < 0.001.^42^ Swayden found a significant difference in patient-perceived time that clinicians spent at the bedside (sit: actual 1.04 min, perceived 5.14 min; stand: actual 1.28 min, perceived 3.44 min; *p* = 0.01 for both comparisons), after excluding outliers.^45^ Two studies found that while patients overestimated encounter duration regardless of posture type, the difference in differences between actual and perceived encounter duration based on posture was not significant.^43,49^

##### Preferences for Posture Type

Four studies reported patient preferences for posture types. Strasser’s evaluation of patients with cancer randomized to successive consultation videos (video A: clinician sitting; video B: clinician standing) revealed no differences in patient preferences.^44^ The follow-up study by Bruera, however, revealed that 50.6% and 17.3% of patients preferred the consultation with the sitting and standing clinicians, respectively, and 32.1% had no preference (*p* < 0.001).^40^ For both studies, the second consultation was rated more favorably regardless of the posture order. Gupta’s survey-based studies of Caucasian and African American/Hispanic patients noted that 60% and 58% of respondents, respectively, indicated that they were most comfortable with sitting (as opposed to standing) clinicians.^52,53^

##### Interaction Quality, Provider Communication, and Provider Compassion

Four studies demonstrated a positive impact of clinician sitting on patient perceptions of quality, communication, and compassion. Orloski found that when compared to encounters involving standing clinicians, sitting at any point during the encounter was associated with improved responses to satisfaction questions, including clinician politeness and the patient feeling cared for, listened to, informed, and given adequate time (*p* < 0.001 for all).^48^ Similarly, Golden noted that frequent sitting (every time or most times) correlated with positive impressions of the clinician (including spent enough time at the bedside, checked for complete understanding, allowed patients to talk without interrupting, and never seemed to be in a rush every single time [*p* < 0.01 for all]).^50^ In analysis of qualitative data, Swayden found that 95% of patients with whom clinicians sat expressed positive comments regarding their care vs. 61% with whom clinicians stood.^45^ Finally, Bruera found that patients viewed seated clinicians more highly and as more compassionate.^40^ Bruera and colleagues noted, however, that patients rated other clinician attributes (e.g., time spent, warmth, patience, respect) as more important than posture when describing their ideal consultation.

Three studies demonstrated either no impact between posture types or a positive impact for standing posture on patient perceptions. Johnson reported no differences in the quality of patient-clinician interactions (e.g., good bedside manner, cared about me, understood my problems) between posture groups.^42^ Similarly, Pattison’s study of nursing leader posture found no differences in patient perceptions of interaction quality (e.g., adequacy of time spent, nurse leader cared about patient) by posture.^49^ Finally, Donovan found that a standing posture (rather than sitting) was associated with higher patient rating of communication skills, specifically for the prompts “my [clinician] told me everything” (*p* = 0.037) and “my [clinician] gave me time to ask questions” (*p* = 0.032) when analyzed based on intention to treat.^41^ Donovan and colleagues noted that while these findings had statistical significance, they were unlikely to have clinical significance.

### Results of Standardized Assessments of Patient Satisfaction

Four studies reported outcomes from standardized patient satisfaction tools. George’s “commit to sit” nursing initiative—in which nurses sat at the bedside for 3–5 min at the beginning of each shift—resulted in improved nurse-patient communication HCAHPS scores from the 4th to the 90th percentile within three years.^46^ Horton’s examination of the effect of implementing a standardized communication intervention (education, laminated card, emphasis on sitting) for hospitalists and internal medicine residents revealed that the proportion of patients answering “yes” to three HCAHPS communication questions increased from 55.5 to 62.8% (*p* = 0.014) over the study period (2012–2014), while control group scores remained stable.^47^ Tackett found that etiquette-based medicine behaviors were associated with higher clinician-specific Press Ganey scores (*p* = 0.019), although the only significant individual behavior was sitting at the bedside (*p* = 0.026).^51^ Finally, Merel found that seated clinicians had a higher proportion of patients who responded “always” to the HCAHPS prompts of “explains things in a way that is easy to understand” and “listens carefully to you;” however, when re-analyzed based on clinician-reported adherence to randomization, these differences were no longer present.^43^

## DISCUSSION

In this systematic review, we evaluated 14 studies that examined hospitalized patient perceptions of clinicians who were seated compared with those who stood. Included studies were noted to be markedly heterogeneous in terms of study design, interventions, measurement types, and outcomes, and we rated most studies as having an elevated risk of bias. Additionally, it is likely that both lower adherence to sitting for those clinicians assigned that posture (within randomized controlled trials) and low frequency of perceived clinician sitting (within observational studies) influenced the results of included studies. Nonetheless, 10 of 14 studies noted some favorable outcome for clinicians who sat when communicating (e.g., perceptions of clinician compassion and time spent, higher scores on satisfaction surveys), three revealed no difference in patient preferences and perceptions, and one indicated higher patient ratings of communication for clinicians who stood. Taken together, these results suggest that a seated clinician posture, as a nonverbal communication technique, may positively impact the patient experience.

What explains the preference for seated clinicians? In the social psychology literature, nonverbal behaviors are inextricably linked with several relational dimensions including hierarchy, status, and power.^54^ Nonverbal cues put forth by clinicians can affect perceptions of their size and power in the patient-clinician relationship.^55^ Because shared decision-making and relationship-centered care have eclipsed historical models of physician-centric, patriarchal care,^56^ it is conceivable that patients desire and expect their clinicians to share power during encounters. One way to even the hierarchy is through eye-level communication. We postulate that this power sharing is particularly important to patients at times of high stress and acuity that are typified by the inpatient hospital environment.

Our findings expand upon prior literature regarding the influence of clinician nonverbal behaviors. Consider the mixed results in the pediatric population. A prospective, randomized trial that compared sitting and standing inpatient pediatric clinician teams showed no differences in actual time spent in the encounter. There were also no significant differences in perceived time spent, nor in “top-box” family experience questions based on clinician posture.^57^ In a study using simulated pediatric patient death scenarios, clinicians who sat while conversing with the parent actor garnered higher rankings from evaluators compared to those who stood.^58^ In another study, children who were shown photographs of physicians in different postures more often preferred the one standing as opposed to stooping at eye level. As noted in our study, preferences for clinician posture were not universal, but rather appear to be dependent on several variables including population, context, and setting.

Clinician adherence to a seated posture was lower among those assigned to sitting compared with adherence to standing posture among those assigned to standing. An understanding of the root causes that lead to standing and/or avoidance of sitting at the bedside are necessary when considering future interventions. Three of the included studies cited clinicians’ reported barriers to sitting.^41,43,50^ Identified barriers included the lack of consistently available seating, seating that is occupied by other objects, discrepancy between self-perceived and actual rates of sitting, a perception of lack of time and/or that the encounter will take longer if the clinician sits, perceived inefficiency from sitting, and concerns over pathogen transmission for patients in contact precautions. Many of these barriers are less relevant in the outpatient clinic setting, which often has dedicated, unobstructed seating that is expected to be used by the clinician.

Questions remain regarding how to encourage clinicians to sit when communicating with patients in the hospital. For those healthcare systems that wish to promote this behavior, it is likely that several changes would be necessary such as adjusting local culture to one that expects clinicians to sit, role modeling patient-centered posture in clinician teams, and eliminating physical barriers (e.g., equipping each patient room with a dedicated form of seating for use by the clinician). Among the studies that appeared to improve and sustain rates of clinician sitting, a combination of specific instruction to sit and provision of dedicated seating seemed to be more effective than either of these interventions in isolation.^43,48^

Our study should be interpreted in the context of several limitations. First, the study is prone to publication bias because we excluded articles not published in English and those limited only to poster presentations. Second, marked heterogeneity among included studies limited our examination; reported patient preference outcome types varied among studies, and at times, clinician posture was only one type of intervention among several bundled interventions related to communication skills. These factors led to a diminished ability to associate interventions with exact outcomes. Because of study heterogeneity, we were unable to conduct a formal meta-analysis. Furthermore, the elevated risk of bias we identified in most included studies creates uncertainty in the presented results. Finally, subjective patient perceptions of clinician time and communication effectiveness are only one type of outcome among many that are important for effective healthcare, and they must be interpreted in the context of other key clinical outcomes.

Our findings carry important clinical implications. At the micro-system level, we believe our results should encourage clinicians to consider eye-level posture with their hospitalized patients, as this approach may yield elevated perceptions of communication effectiveness and patient-clinician rapport. Effective communication addresses multiple components of the Quintuple Aim of Healthcare^59^ and may have benefits including improved patient care outcomes (population health), enhanced care experience, and perhaps reduced healthcare clinician burnout. At the macro-system level, hospitals and practices may consider committing resources to support relationship-centered communication. These resources may take the form of architectural design to ensure availability of unobstructed seating in hospital and ED rooms or educational and cultural change campaigns to encourage effective verbal and nonverbal communication techniques.

In conclusion, when compared to standing, eye-level communication by clinicians may carry benefit, although the magnitude and types of benefits remain unclear given the heterogeneity of study protocols and the generally high risk of bias. A more robust randomized clinical trial, aimed at removing barriers to sitting and elucidating factors that drive patients’ preferences, is necessary to fully understand the impact of posture on patient outcomes. In the meantime, given its relative ease and potential for benefit, clinicians should consider positioning themselves at eye level when speaking with their hospitalized patients.

## Supplementary Information

Below is the link to the electronic supplementary material.

Supplementary file1 (DOCX 21.5 KB)

Supplementary file2 (DOCX 17.8 KB)

Supplementary file3 (DOCX 18.4 KB)

Supplementary file4 (DOCX 26.0 KB)

Supplementary file5 (DOCX 23.2 KB)

## Data Availability

Data are available from the corresponding author upon reasonable request.

## Declarations:

### Registration and Protocol:

We registered our protocol for this review with PROSPERO, the international prospective register of systematic reviews, on 20 August 2020 (CRD42020199817). The protocol is available online at https://www.crd.york.ac.uk/prospero/display_record.php?RecordID=199817. Final update submitted on 30 October 2023.
