# Supplementary material for: Effect of Clinician Posture on Patient Perceptions of Communication in the Inpatient Setting: A Systematic Review
Source: J Gen Intern Med. 2024 Jul 17;39(16):3290–8. doi: 10.1007/s11606-024-08906-4 (PMC11618274; doi:10.1007/s11606-024-08906-4)
Supplement: Supplementary file 1 — Supplementary file1 (DOCX 21.5 KB) [file 11606_2024_8906_MOESM1_ESM.docx]

**Appendix 1**

Systematic Review Search Report: Clinician Posture at the Bedside

**Whitney A. Townsend, MLIS**

**Tools & Techniques from the literature**

*SR Accelerator Polyglot search translation tool*

Clark JM, Sanders S, Carter M, et al. Improving the translation of search strategies using the Polyglot Search Translator: a randomized controlled trial. *J Med Libr Assoc*. 2020;108(2):195‐207. doi:10.5195/jmla.2020.834

<https://www.ncbi.nlm.nih.gov/pubmed/32256231>

*Endnote deduplication technique*

Bramer WM, Giustini D, de Jonge GB, Holland L, Bekhuis T. De-duplication of database search results for systematic reviews in EndNote. *J Med Libr Assoc.* 2016;104(3):240‐243. doi:10.3163/1536-5050.104.3.014

<https://www.ncbi.nlm.nih.gov/pubmed/27366130>

**Databases**

- Medline via Ovid (Ovid MEDLINE(R) and Epub Ahead of Print, In-Process & Other Non-Indexed Citations, Daily and Versions(R))
- Embase.com (including Embase Classic)
- Scopus.com
- Web of Science Core Collection (SCI-EXPANDED, SSCI, A&HCI, CPCI-S, CPCI-SSH, BKCI-S, BKCI-SSH, ESCI, CCR-EXPANDED)
- CINAHL Complete (Ebsco)
- PsycInfo (Ebsco)

**Initial Search (1 October 2020)**

- All searches run from database inception to **1 October 2020**, unless otherwise noted
- All searches run without limits unless otherwise noted
- Original Ovid Medline search strategy was translated and adapted to other databases using <http://sr-accelerator.com/#/polyglot> and the searcher's discretion.
- **Total results before deduplication: 4591 results**
- **Total results after modified Bramer method Endnote deduplication: 2476 (2115 duplicates removed)**

**INITIAL SEARCH NOTES:**

Search strategies were tested against a provided set of relevant citations, and found all. To find the sentinel articles in PubMed, use this search string:

17846090[uid] OR 27378679[uid] OR 15904751[uid] OR 21719234[uid] OR 23423452[uid]

For Ovid Medline, use:

("17846090" or "27378679" or "15904751" or "21719234" or "23423452").ui.

**Ovid Medline (693 on 1 October 2020)**

(((physician* or provider* or doctor* or nurse or nurses or "physician assistant" or "physician assistants" or clinician or clinicians or bedside) adj3 (posture or sitting or standing or sit or sits or stand or stands or "eye level" or "eye-level" or eyelevel)) or ("etiquette-based" or "etiquette based" or "bedside etiquette")).mp.

**Embase (907 on 1 October 2020)**

(((physician* OR provider* OR doctor* OR nurse OR nurses OR "physician assistant" OR "physician assistants" OR clinician OR clinicians OR bedside) NEAR/3 (posture OR sitting OR standing OR sit OR sits OR stand OR stands OR "eye level" OR eye-level OR eyelevel)) OR (etiquette-based OR "etiquette based" OR "bedside etiquette")):ti,ab,de,tn

**Scopus (950 on 1 October 2020)**

(TITLE-ABS-KEY((physician* OR provider* OR doctor* OR "physician assistant" OR "physician assistants" OR clinician OR clinicians OR bedside) W/3 (posture OR sitting OR standing OR sit OR sits OR stand OR stands OR "eye level" OR "eye-level" OR eyelevel))) OR (TITLE-ABS-KEY("etiquette-based" OR "etiquette based" OR "bedside etiquette"))

**Web of Science Core Collection (980 on 1 October 2020)**

TS=((((physician* OR provider* OR doctor* OR nurse OR nurses OR "physician assistant" OR "physician assistants" OR clinician OR clinicians OR bedside) NEAR/3 (posture OR sitting OR standing OR sit OR sits OR stand OR stands OR "eye level" OR eye-level OR eyelevel)) OR (etiquette-based OR "etiquette based" OR "bedside etiquette")))

**CINAHL (853 on 1 October 2020)**

TI ( (((physician* OR provider* OR doctor* OR nurse OR nurses OR "physician assistant" OR "physician assistants" OR clinician OR clinicians OR bedside) N3 (posture OR sitting OR standing OR sit OR sits OR stand OR stands OR "eye level" OR eye-level OR eyelevel)) OR (etiquette-based OR "etiquette based" OR "bedside etiquette")) ) OR AB ( (((physician* OR provider* OR doctor* OR nurse OR nurses OR "physician assistant" OR "physician assistants" OR clinician OR clinicians OR bedside) N3 (posture OR sitting OR standing OR sit OR sits OR stand OR stands OR "eye level" OR eye-level OR eyelevel)) OR (etiquette-based OR "etiquette based" OR "bedside etiquette")) )

**PsycInfo (208 on 1 October 2020)**

TI ( (((physician* OR provider* OR doctor* OR nurse OR nurses OR "physician assistant" OR "physician assistants" OR clinician OR clinicians OR bedside) N3 (posture OR sitting OR standing OR sit OR sits OR stand OR stands OR "eye level" OR eye-level OR eyelevel)) OR (etiquette-based OR "etiquette based" OR "bedside etiquette")) ) OR AB ( (((physician* OR provider* OR doctor* OR nurse OR nurses OR "physician assistant" OR "physician assistants" OR clinician OR clinicians OR bedside) N3 (posture OR sitting OR standing OR sit OR sits OR stand OR stands OR "eye level" OR eye-level OR eyelevel)) OR (etiquette-based OR "etiquette based" OR "bedside etiquette")) )

***Search updates used the same search strings as noted above for the initial search.***

**Search Update (14 February 2022): 346 new unique citations**

- Ovid Medline - 728 - new unique: 40
- Embase - 949 - new unique: 41
- Scopus - 1,021 - new unique: 49
- Web of Science - 1,148 - new unique: 173
- CINAHL - 901 - new unique: 37
- PsycInfo - 220 - new unique: 6

**Search Update (2 May 2023): 254 new unique citations**

- Ovid Medline - 784 - new unique: 64
- Embase - 1044 - new unique: 28
- Scopus - 1112 - new unique: 58
- Web of Science - 1265 - new unique: 74
- CINAHL - 942 - new unique: 27
- PsycInfo - 230 - new unique: 3
