# Supplementary material for: Effect of Clinician Posture on Patient Perceptions of Communication in the Inpatient Setting: A Systematic Review
Source: J Gen Intern Med. 2024 Jul 17;39(16):3290–8. doi: 10.1007/s11606-024-08906-4 (PMC11618274; doi:10.1007/s11606-024-08906-4)
Supplement: Supplementary file 2 — Supplementary file2 (DOCX 17.8 KB) [file 11606_2024_8906_MOESM2_ESM.docx]

**Appendix 2**

Study Data Extraction Template Elements

1. **General Information**
   1. Study ID
   2. Study Title
   3. Is Conference Abstract? (Yes/ No)
   4. Country in which study conducted: United States, UK, Canada, Australia, Other (fill in)
   5. General Notes (study-specific research notes)

# Study Characteristics – Methods

- 1. Study Design: Case-control, Cohort, Randomized Controlled Trial, Cross-sectional, Other (fill in)
  2. Study Aim (fill in)
  3. Outcome Description
  4. Outcome Results
  5. Intervention/Control

# Study Characteristics – Participants

- 1. Population Description (Include Setting)
  2. Inclusion Criteria
  3. Exclusion Criteria
  4. Total Number of Participants
