# Supplementary material for: Effect of Clinician Posture on Patient Perceptions of Communication in the Inpatient Setting: A Systematic Review
Source: J Gen Intern Med. 2024 Jul 17;39(16):3290–8. doi: 10.1007/s11606-024-08906-4 (PMC11618274; doi:10.1007/s11606-024-08906-4)
Supplement: Supplementary file 3 — Supplementary file3 (DOCX 18.4 KB) [file 11606_2024_8906_MOESM3_ESM.docx]

**Appendix 3**

Abstract-only publications identified by this systematic review.

| **Author, Year** | **Journal** |
| --- | --- |
| Bruera, 2006 | *Psycho-Oncology* |
| Donovan, 2019 | *Journal of General Internal Medicine* |
| Gambarota, 2005 | *Annals of Emergency Medicine* |
| Kwan, 2015 | *Journal of General Internal Medicine* |
| Nissley, 2016 | *Journal of Cardiac Failure* |
| Orloski, 2016 | *Annals of Emergency Medicine* |
| Tackett, 2011 | *Journal of General Internal Medicine* |
| Wadsworth, 2017 | *Journal of Obstetric Gynecologic and Neonatal Nursing* |
| Wangcheng, 1992 | *Clinical Research* |
| Zehner, 2016 | *Journal of Clinical Oncology* |
