# Supplementary material for: Effect of Clinician Posture on Patient Perceptions of Communication in the Inpatient Setting: A Systematic Review
Source: J Gen Intern Med. 2024 Jul 17;39(16):3290–8. doi: 10.1007/s11606-024-08906-4 (PMC11618274; doi:10.1007/s11606-024-08906-4)
Supplement: Supplementary file 4 — Supplementary file4 (DOCX 26.0 KB) [file 11606_2024_8906_MOESM4_ESM.docx]

**Appendix 4**

Additional Information on Included Studies

| **Reference** | **Journal** | **Study Location** | **Patient Demographics** |
| --- | --- | --- | --- |
| *Randomized Controlled Trials* | | | |
| Bruera, 2007^39^ | *Palliat Med* | Houston, TX | - 16 years and older - Cognitively intact - Palliative care/Rehabilitation medicine - Inpatients or outpatients - Metastatic disease (no longer curable) |
| Donovan, 2020^40^ | *Patient Educ Couns* | Pittsburgh, PA | - Internal Medicine residents |
| Johnson, 2008^41^ | *Ann Emerg Med* | Rochester, MN | - Adults presenting to the ED |
| Merel, 2016^42^ | *J Hosp Med* | Seattle, WA | - Adults consenting to own medical care - Newly admitted or transferred from ICU - English speaking |
| Strasser, 2005^43^ | *J Pain Symptom Manage* | Houston, TX | - 16 years and older - Cognitively intact - Palliative care/Rehabilitation medicine - Inpatients or outpatients - Advanced cancer |
| Swayden, 2012^44^ | *Patient Educ Couns* | Kansas City, KS | - Post-op elective neurosurgical - *Not collected*: age, education, gender |
| *Quasi-Experimental Studies* | | | |
| George, 2018^45^ | *Crit Care Nurse* | Houston, TX | - Hospitalized (no further details) |
| Horton, 2017^46^ | *Am J Med Qual* | Salt Lake City, UT | - Admitted adults, *except:* - OB/GYN, rehabilitation, psychiatric |
| Orloski, 2019^47^ | *J Patient Exp* | Philadelphia, PA | - Patients discharged home after ED visit - 63% female - 65% between 26 and 65 years of age |
| Pattison, 2017^48^ | *J Nurs Care Qual* | Kalamazoo, MI | - Adult - Inpatient (Medical-surgical) |
| *Observational (Cross-Sectional Studies)* | | | |
| Golden, 2022^49^ | *J Gen Intern Med* | Baltimore, MD | - All admitted to medicine service |
| Tackett, 2013^50^ | *J Gen Intern Med* | Baltimore, MD | - All discharged |
| Gupta, 2015A^51^ | *J Med Pract Manag* | Dallas, TX | - Caucasian patients admited to medicine service |
| Gupta, 2015B^52^ | *J Med Pract Manag* | Dallas, TX | - Black or Hispanic patients admitted to medicine service |
| *Note: ED = emergency department; ICU = intensive care department; OB/GYN = obstetrics/gynecology* | | | |
